# Popliteal artery thrombosis following total knee arthroplasty: A preventable complication with surveillance

**DOI:** 10.4103/0019-5049.76575

**Published:** 2011

**Authors:** Vijaya Pant, Preety Mittal Roy, Jyotirmoy Das, Umesh Deshmukh, Prem Kakar

**Affiliations:** Department of Anaesthesia, Fortis Hospital, Shalimar Bagh, New Delhi, India; 1 Department of Anaesthesia Primus Superspeciality Hospital, New Delhi, India; 2 Department of Anaesthesiology, Fortis Hospital, Shalimar Bagh, New Delhi, India

Sir,

We are reporting a case of popliteal artery thrombosis after total knee arthroplasty (TKA).

A 60-year-old ASA Grade II hypertensive female patient was admitted for bilateral total knee arthoplasty. Surgery was conducted under combined spinal and epidural anaesthesia and Propofol infusion. Tourniquet time was 56 min on right side and 66 min on left side with a pressure of 300 mmHg. Intraoperative period was uneventful. Postoperatively patient was shifted to the intensive care unit (ICU) as per hospital protocol for pain management and monitoring where she remained comfortable and stable. Epidural infusion (Bupivacaine 0.125% and Clonidine 2 *μ*g/ml) was started at the rate of 6 ml per hour. On examination the left dorsalis pedis and posterior tibial artery pulsation were absent with normal left femoral artery pulsation. Toes on the left leg were cooler than on the right and capillary refill time was prolonged. On Colour Doppler examination no flow was detected at the level of the knee and below. Vascular surgeon was consulted and patient was managed by emergency thrombectomy under epidural anaesthesia. After completion of surgery epidural catheter was removed in the operation theatre. In the postoperative period Aspirin 150 mg daily and Clopidogrel 150 mg twice daily was started immediately. Enoxaparin was started 2 h postoperatively and Warfarin was added 48 h later to achieve a target international normalized ratio (INR) of 2 to 3. Patient was discharged with no further complications.

On reviewing the literature, we found that arterial vascular injury is a rare complication of TKA (incidence 0.03–0.17%).[1,2] Most of these vascular complications involve thrombosis, atherosclerotic occlusion, or direct sharp trauma. Preoperative risk factors for arterial injury are history of intermittent claudication, ischaemic rest pain, arterial ulcers, absence of distal pulses, popliteal aneurysm, previous arterial reconstruction and arterial calcification on plain radiographs.[3]

Patient should be evaluated for these risk factors in the preoperative assessment and documentation done accordingly. If necessary a vascular surgeon should be consulted. However, no guideline was found in the literature contraindicating arthroplasty in the presence of risk factors. Rusch and others,[4] have advocated that in the presence of risk factors surgery without tourniquet may be beneficial in prevention of vascular complication.

To conclude, a high index of suspicion for postoperative arterial complication should be maintained in the form of monitoring peripheral pulses, limb colour (pallor), temperature and if necessary Doppler assessment of the limb to prevent this limb-threatening complication.
